# Estimation of seasonal influenza disease burden using sentinel site data in Pakistan 2017–2019: A cross‐sectional study

**DOI:** 10.1111/irv.13125

**Published:** 2023-03-21

**Authors:** Muhammad Salman, Nazish Badar, Aamer Ikram, Nadia Nisar, Umer Farooq

**Affiliations:** ^1^ Public Health Laboratories Division National Institute of Health Islamabad Pakistan; ^2^ Executive Director National Institute of Health Islamabad Pakistan; ^3^ National Agricultural Research Center Islamabad Pakistan

**Keywords:** disease burden, influenza, Pakistan

## Abstract

**Background:**

The influenza A(H1N1)pdm09 pandemic highlighted the need for reliable disease burden estimation from low‐ and middle‐income countries like Pakistan. We designed retrospective age‐stratified estimation of influenza‐related severe acute respiratory infections (SARIs) incidence in Islamabad Pakistan 2017–2019.

**Materials and Methods:**

The catchment area was mapped on SARI data from one designated influenza sentinel site and other healthcare facilities in the Islamabad region. The incidence rate was calculated as per 100,000 for each age group with 95% confidence interval.

**Results:**

The catchment population for the sentinel site was 0.7 million against the total denominator of 1.015 million, and incidence rates were adjusted. During January 2017 to December 2019, among 13,905 hospitalizations, 6715 (48%) patients were enrolled; 1208 of these (18%) were positive for influenza. During 2017, influenza A/H3 dominated with 52% detections followed by A(H1N1)pdm09 (35%) and influenza B (13%). Furthermore, elderly 65+ years age group had highest hospitalizations and influenza positive. The incidence rates of all cause respiratory and influenza‐related SARI were highest among children >5 years; highest incidence was found in 0 to 11 month/year group with 424/100,000 cases and lowest in 5–15 years 56/100,000. The estimated average annual influenza‐associated hospitalization percentage was 29.3% during the study period.

**Conclusion:**

Influenza accounts for a significant proportion of respiratory morbidity and hospitalization. These estimates would enable governments for evidence‐based decisions and priority allocation of health resources. It is necessary to test for other respiratory pathogens for more clear disease burden estimation.

## 1 INTRODUCTION

Influenza virus infections pose substantial risk of morbidity and mortality globally, particularly in young children and elderly people.^1^ The World Health Organization (WHO) estimates that the influenza virus infection alone results in between 290,000 and 650,000 annual deaths,^2^ with 36% of these deaths taking place in low‐ and middle‐income countries (LMICs) like Pakistan. The epidemiology and disease burden of influenza is poorly understood in the region. In Pakistan, with a temperate climate and a population of about 22.5 billion people influenza virus peaks during winter months (October–February).^3^ To assess the severity of the influenza disease, it is crucial to track influenza incidence and hospitalization.

According to WHO, there is need for influenza virus associated disease burden estimates, particularly from low‐ and middle‐income nations.^4^ With the aid of these projections, governments would be able to allocate limited resources and devise intervention efforts to lessen the impact and spread of the disease. National estimates would also help to clarify how widespread influenza‐associated illness is and would help to guide global public health priorities.

Severe acute respiratory infections (SARIs), including influenza, constitute a major cause of morbidity and mortality globally.^5^ Influenza A(H1N1)pdm09 pandemic emphasized reliable influenza disease burden estimates from LMICs like Pakistan to better recognize the impact of this vaccine preventable disease. Although global data on SARI is present, reliable information on disease burden estimates due to Influenza are still not available for Pakistan.

Seasonal fluctuations in influenza virus infection are known, and vaccination campaigns are conducted in many countries at the beginning of influenza season to reduce morbidity and mortality.^6^ However, in Pakistan, immunization against influenza is not being given high attention. Currently, the Ministry of National Health Services, Regulations and Coordination (MoNH&RC) advises that high‐risk populations, such as healthcare workers, pregnant women, and persons with chronic health conditions, receive an annual influenza vaccination. Children below 5 years and adults 65 years and above, the seasonal influenza vaccine is recommended but MoNH&RC did not include in routine immunization program for these age groups.^7^ Governments will be able to make evidence‐based decisions for preventative and control measures, such the adoption of the seasonal influenza vaccine in public health programs, after having these age‐specific burden estimates. We designed a retrospective study to generate a preliminary estimate for age‐specific incidence of influenza in the Islamabad region during 2017–2019.

## 2 METHODS

### 2.1 Study population

The National Influenza Center (NIC) of the NIH in Islamabad serves as the national coordination hub for the laboratory‐based influenza virus surveillance network that the National Institute of Health (NIH) developed.^8^ Cases were enrolled in a cross‐sectional study to calculate the burden of influenza with SARIs. We selected one of our influenza surveillance sentinel site Federal Government Services Hospital (FGSH) at Islamabad during 2017–2019 for burden of disease estimation.

The catchment area of the sentinel site at FGSH overlaps with three major hospitals: Pakistan Institute of Medical Sciences (PIMS) with associated Children's Hospital, Capital Hospital, and finally, a major private sector hospital, Shifa International Hospital (Table 1). Our site, FGSH, covers 80% of federal employees and their families along with 20% of nonentitled patients (no private health care coverage). PIMS is the largest public sector hospital in Islamabad which provides healthcare to 40% of the city's population and 60% is from outside Islamabad. Children's Hospital treats 60% of Islamabad's under 16 population while 40% of patients come from outside the Islamabad region. The Capital Hospital owned and provides healthcare to mostly Capital Development Authority (CDA) employees. The Shifa International Hospital is the largest private sector hospital in Islamabad that provides healthcare to 40% of the Islamabad population while the remaining 60% of patients are from outside Pakistan.

**TABLE 1:** The catchment area of sentinel site at FGSH.

Sr #	Major hospitals in Islamabad	No. of beds	Sector owned	Population coverage	SARI data availability	Availability of morbidity and outcome data
i	FGSH	545	Public	80% federal employees and their families 20% other or nonentitled	SARI designated sentinel site	Yes (sentinel site)
ii	PIMS	846	Public	40% of Islamabad population 60% outside Islamabad	Yes	No or limited sharing
	Children Hospital	254	Public	60% of Islamabad population 40% outside Islamabad	Yes	
iii	Capital Hospital	270	CDA owned	80% of CDA employees and families 20% other or nonentitled	No	
iv	Shifa International Hospital	550	Private	40% of Islamabad population 60% outside Islamabad	No	

### 2.2 Case definition for sample collection

According to the WHO prescribed case definition for screening hospitalized patients with SARI: sudden onset of fever (>38°C), cough/sore throat that required hospital admission within 10 days termed as SARI, and children above 5 years with difficulty breathing or clinically suspected pneumonia with increased respiratory rate requiring hospitalization.^9^

### 2.3 Ethical clearance

The Internal Review Board of Pakistan NIH gave its approval to the SARI data collection and surveillance. Each participant provided formal written or verbal consent; nevertheless, patient identities have never been revealed. The institutional board authorized this kind of consent after being informed of the particular needs related to the study setting. The data form had a checkbox for recording the consent gathering process.

### 2.4 Laboratory testing

Respiratory specimens were tested for influenza viruses using real‐time reverse‐transcription polymerase chain reaction (rRT‐PCR) (CDC protocol). From 2017 to 2019, we collected five to 10 nasopharyngeal swabs per week.^10^ The data were gathered all year round to show influenza virus circulation pattern. By influenza virus type (A or B), influenza A subtype (H1N1pdm09 or H3N2), and overall number of specimens analyzed, we were able to determine the number of specimens that tested positive each week. Viral surveillance data were stratified by age group and utilized in models due to the lack of viral surveillance data for the age groups in this investigation.

### 2.5 Disease burden estimation

**FIGURE 1:**
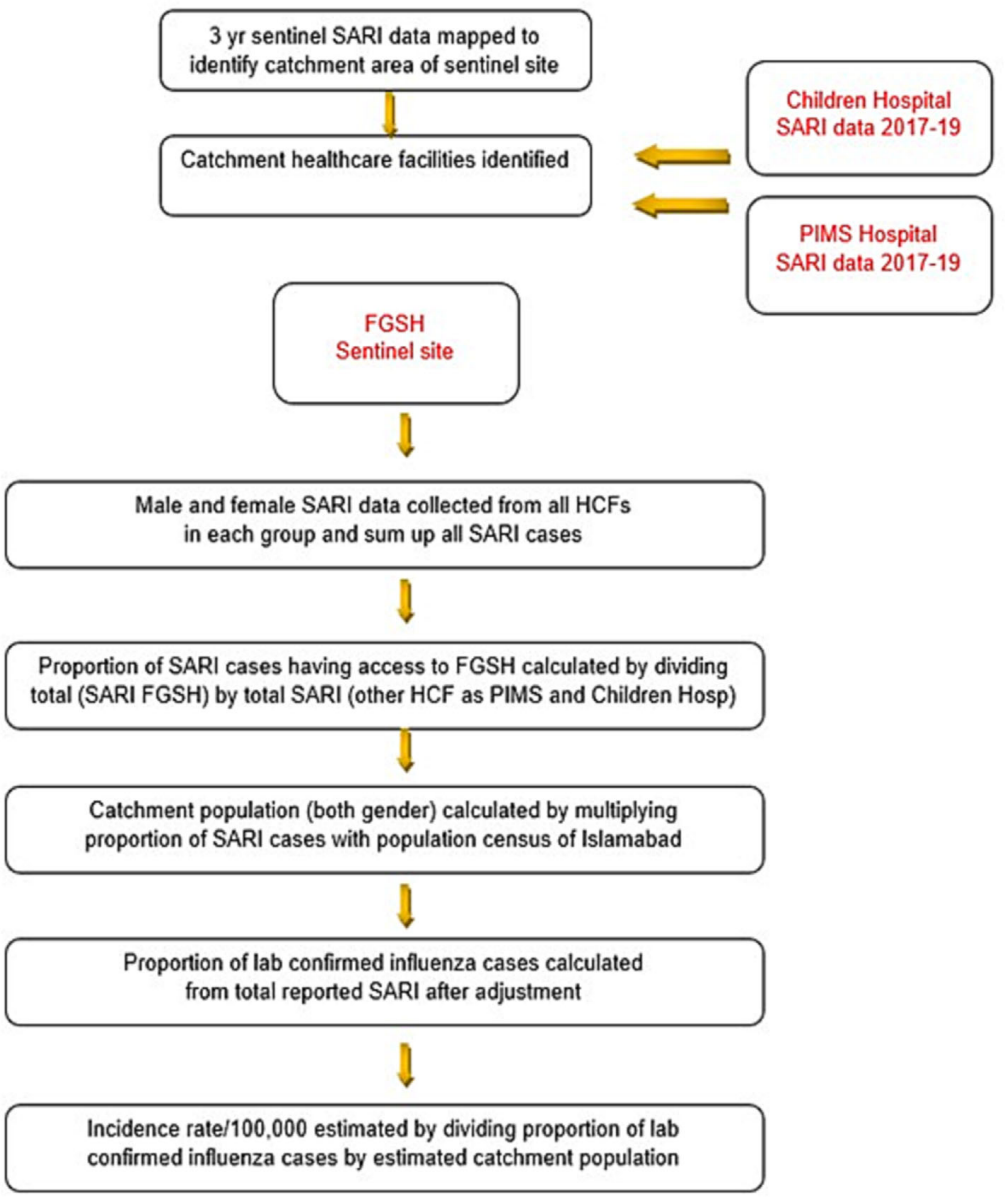
Methodology for disease burden estimation.

**FIGURE 2:**
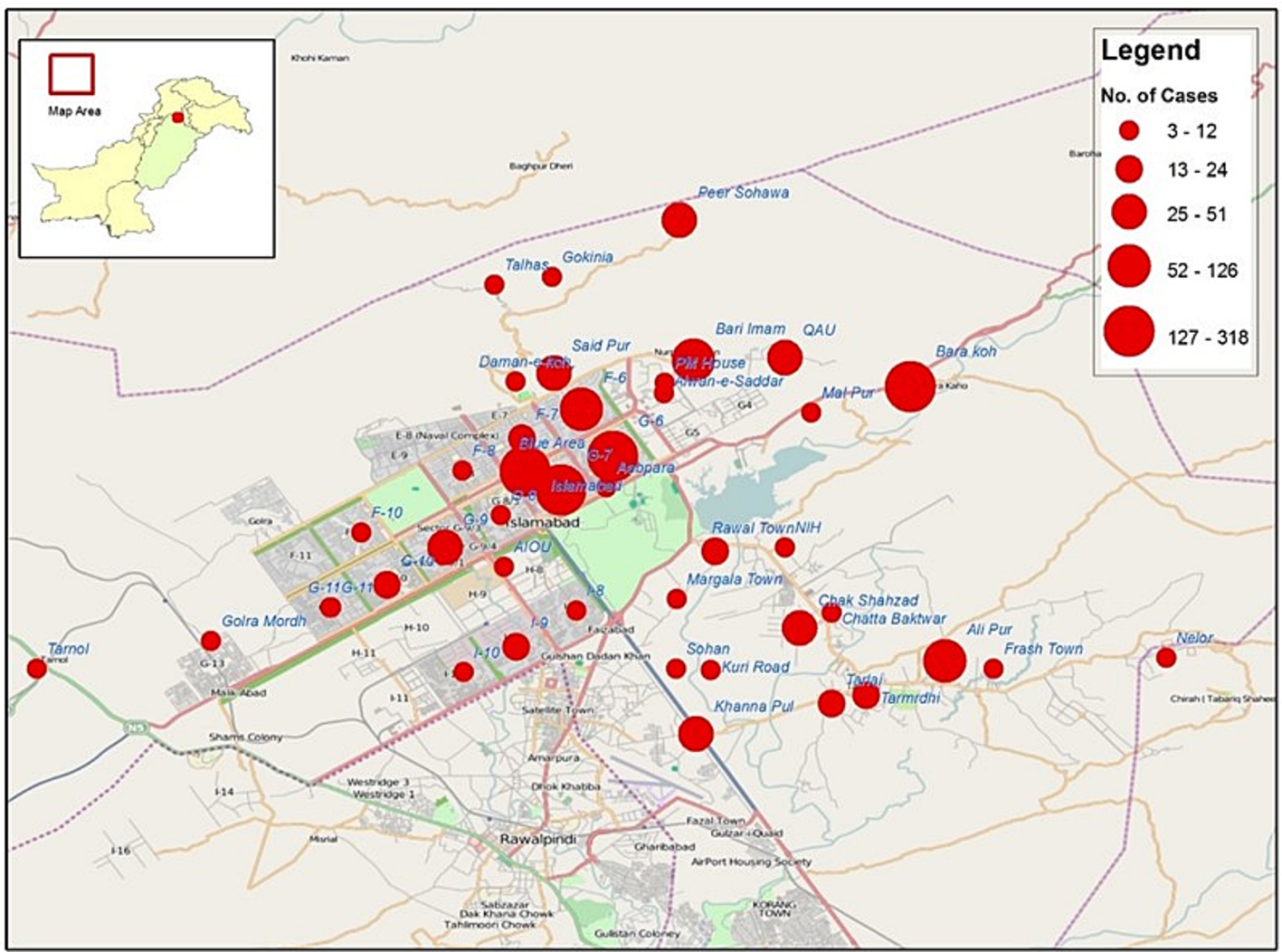
Geographical location of SS (red dot) at FGSH in capital Islamabad.

Incidence rates were estimated according to WHO manual for disease burden estimation of influenza (Figure 1).^4^ The catchment area was mapped (GIS) based on SARI data collected from designated influenza sentinel site and other healthcare facilities in the Islamabad region. The catchment population was estimated based on data obtained from FGSH, all SARI cases reported from both urban and rural areas of Islamabad region were mapped, and all cases outside the catchment area were excluded (Figure 2).

From the calculated number of influenza‐associated SARI cases from the designated sentinel hospital, the proportion of lab confirmed influenza cases was inferred for the catchment population (denominator) after adjustment of sampling fraction. The lab confirmed influenza cases in the catchment population was estimated by adjusting the proportion for the catchment population. The proportion of SARI‐associated hospitalizations reported outside the catchment area was determined for the total population under demographic surveillance and incidence rates were adjusted accordingly. To estimate the proportion of influenza SARI cases, the numerator is total positive cases annually and denominator is total hospitalized cases annually. The incidence rate was calculated as per 100,000 population for each age group with 95% confidence interval. The cases were stratified into different age groups on the basis of age and compared through analysis of variance (ANOVA) under cross‐sectional study.

## 3 RESULTS

### 3.1 Catchment population/demographics

The catchment population for the sentinel site is 7,000,000 against the total denominator of 1,200,000 and incidence rates were accordingly adjusted. During January 2017 to December 2019, among 9775 hospitalizations, 6610 (67%) patients were enrolled; 1617 of these (24%) were positive for influenza. In 2017, FGSH reported (55%) of SARI cases in catchment area. The share of other major healthcare facilities as PIMS was (10%) while Children Hospital was (27%). Due to enhanced SARI surveillance in 2018, the FGSH reported (69%) of SARI cases in catchment area while PIMS reported (24%) and Children Hospital (7%), respectively. In 2019, FGSH reported (61%) of SARI cases in catchment area while PIMS reported (34%) and Children Hospital (5%), respectively.

### 3.2 Influenza subtype distribution

**FIGURE 3:**
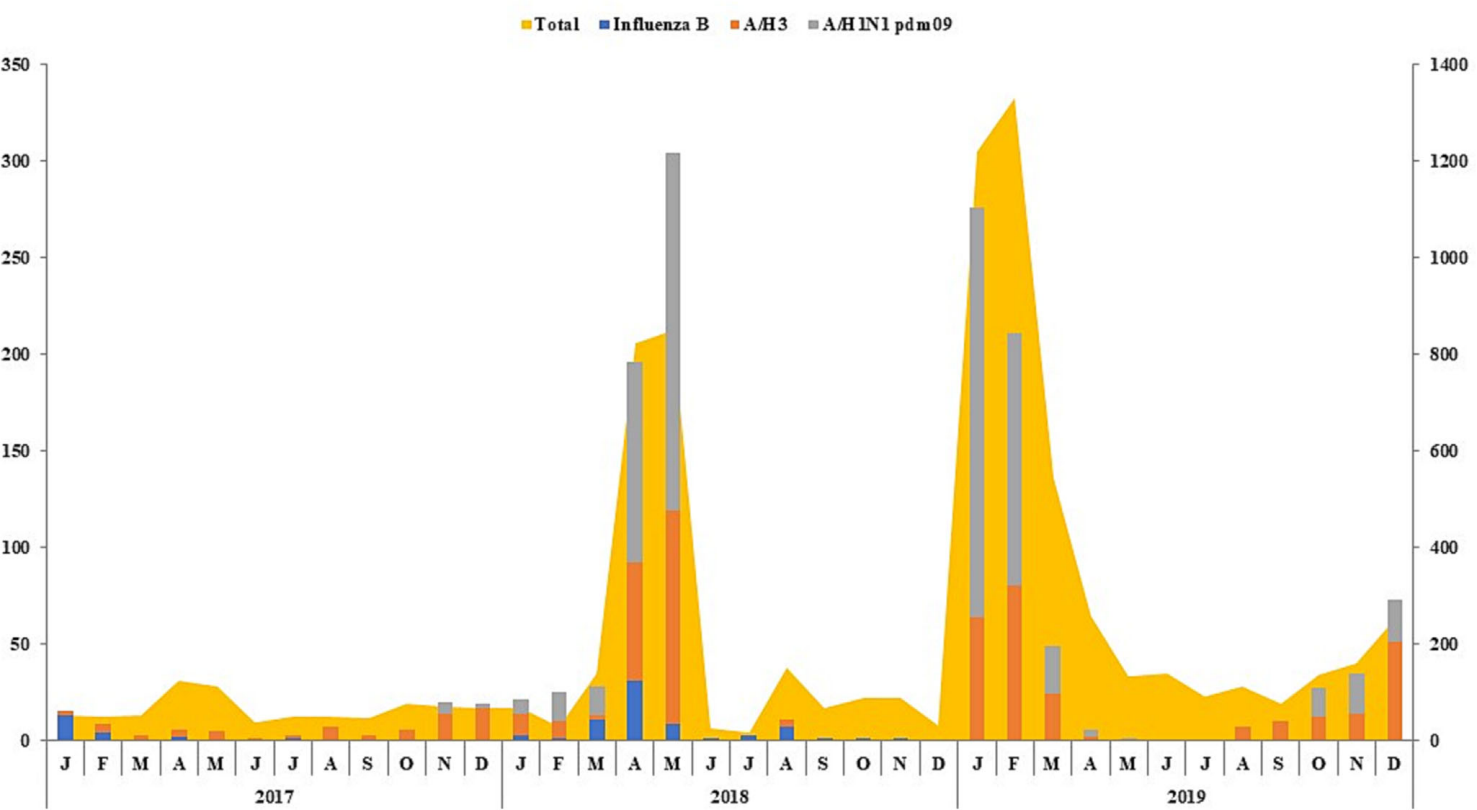
Circulation pattern of influenza viruses 2017–2019.

During 2017, 1850 SARI cases were reported. Samples were obtained from 422 (22%) SARI cases and tested (Figure 3). Of those, 18% (78) were influenza positive. Virus subtyping found that seasonal influenza A/H3 dominated with 40 (52%) detection rate followed by influenza A(H1N1)pdm09 27 (35%) and influenza B 11 (13%).

In comparison, during 2018, as a result of enhanced surveillance at this site, 3659 SARI cases were reported. Samples were collected from 51% (1881) of SARI cases. 26% (488) cases were influenza positive; influenza A(H1N1)pdm09 subtype was detected 332(68%) more frequently followed by A/H3 96 (19.5%) and influenza B 60 (12%).

However, in 2019, 4266 SARI cases were reported. Samples were collected from 57% (2457) of SARI cases. 38% (935) cases were influenza positive; influenza A(H1N1)pdm09 subtype was dominant 411 (44%) followed by influenza B 346 (37%) and A/H3 178 (19%). Hospitalization rate due to influenza B virus was two times higher during 2019 as compared with 2017 and 2018 Figure 3.

### 3.3 Incidence rates by age groups

In comparison with 2017–2018, the proportion of influenza positive hospitalized patients who also had an acute medical condition increased significantly in 2019 (*p* = 0.0001). The age of the patients ranged from 0 to 90 years with a mean age of 32 (SD ± 22) years; most of those tested positive was from the age group 15–50 years (27%) (Table 2).

**TABLE 2:** Total number of SARI from catchment area of sentinel site.

Age groups	2017						2018						2019					
	SARI cases			Total SARI cases	Total suspected SARI cases	Total influenza‐associated SARI cases *n* (%)	SARI cases			Total SARI cases	Total suspected SARI cases	Total influenza‐associated SARI cases *n* (%)	SARI cases			Total SARI cases	Total suspected SARI cases	Total influenza‐associated SARI cases *n* (%)
	FGSH	PIMS	Children hospital				FGSH	PIMS	Children hospital				FGSH	PIMS	Children hospital			
<1	90	‐	97	187	54	5 (9%)	402	‐	209	608	56	19 (34%)	486	15	239	1040	102	37 (36%)
1–2	109	‐	13	122	41	3 (7%)	117	‐	112	226	36	11 (31%)	128	21	123	573	80	23 (29%)
3–5	258	‐	20	278	45	4 (9%)	66	‐	345	411	205	33 (16%)	73	16	375	770	266	51 (19%)
5–15	304	‐	26	330	52	5 (10%)	112	‐	86	198	146	30 (21%)	124	41	95	560	201	36 (18%)
15–50	251	353	‐	604	123	18 (15%)	299	91	‐	1425	891	249 (28%)	329	109	14	752	1040	592 (27%)
50–64	72	127	‐	199	61	29 (47%)	48	46	‐	791	358	97 (27%)	53	55	11	470	471	122 (26%)
>65	25	105	‐	130	46	12 (26%)	32	17	‐		189	49 (26%)	35	21	9	101	297	74 (25%)

*Note*: Red colour shows that the highest percentage of influenza‐associated SARI cases were in elderly group 50–64 years of age followed by >65 years of age during 2017. Green colour shows that the highest percentage of influenza‐associated cases was in children <1 year of age group during 2018–2019.

Over a 3‐year period, the rates of influenza‐related hospitalization were highest among those aged above 65 years followed by children under 5 years of age and lowest among patients 5–15 years. The estimated average annual influenza‐associated hospitalization percentage was 29.3% during the study period (Table 3). During these years, male patients reported more frequently (55.6%) than females across all age groups. Furthermore, during the study period, the 16–49 year age group had the highest number of hospitalized SARI and influenza positive cases. The incidence rates of all cause respiratory and influenza‐related SARI were highest among children under 5 years during 2017–2019.

**TABLE 3:** Comparison of influenza positivity among age groups, 2017–2019.

Year	Age group (years)	Severe acute respiratory infection hospitalization	Annual influenza positivity	
			n	%
2017	<1	54	9	16.7%
	<2	41	4	9.8%
	1–<5	45	4	8.9%
	5–<15	52	5	9.6%
	15–<50	123	18	14.6%
	50–<65	61	29	47.5%
	≥65	46	12	26.1%
	Total	422	81	19.2%
2018	<1	56	19	33.9%
	<2	36	11	30.6%
	1–<5	205	33	16.1%
	5–<15	146	30	20.5%
	15–<50	891	252	28.3%
	50–<65	358	125	34.9%
	≥65	189	77	40.7%
	Total	1881	547	29.1%
2019	<1	102	37	36.3%
	<2	80	23	28.8%
	1–<5	266	51	19.2%
	5–<15	201	36	17.9%
	15–<50	1040	592	56.9%
	50–<65	471	148	31.4%
	≥65	297	105	35.4%
	Total	2457	992	40.4%
2017–2019	<1	212	65	30.7%
	<2	157	38	24.2%
	1–<5	516	88	17.1%
	5–<15	499	71	14.2%
	15–<50	2054	668	32.5%
	50–<65	890	302	33.9%
	≥65	532	194	36.5%
	Total	4860	1426	29.3%

In 2017, the estimated incidence rates of influenza‐related SARI in different age groups were highest for 0–1 year age group with 289/100,000 cases (95% Cl: 259–319) followed by 234/100,000 in age group 1–2 years (95% Cl: 214–264) while lowest in 25/100,000 in age group 5–15 years (95% Cl: 15–35) (Table 4).

**TABLE 4:** Incidence rate/100,000 population of influenza‐related SARI cases: 2017–2019.

Age groups	2017						2018						2019					
	Suspected SARI cases	Influenza‐associated SARI cases	Total population	Proportion of patents admitted	Estimated catchment population	Influenza‐associated SARI HR per 100,000 population	Suspected SARI cases	Influenza‐associated SARI cases	Total population	Proportion of patents admitted	Estimated catchment population	Influenza‐associated SARI HR per 100,000 population	Suspected SARI cases	Influenza‐associated SARI cases	Total population	Proportion of patents admitted	Estimated catchment population	Influenza‐associated SARI HR per 100,000 population
		A	B	C	D = BXC	A/DX100,000		A	B	C	D = BXC	A/DX100,000		A	B	C	D = BXC	A/DX100,000
<1	54	9	18,706	0.17	3118	289	56	19	21,194	0.34	7191	264	102	37	36,401	0.36	13,204	280
1–2	41	4	17,511	0.10	1708	234	36	11	23,115	0.31	7063	156	80	23	43,015	0.29	12,367	186
3–5	45	4	62,502	0.09	5556	72	205	33	82,503	0.16	13,281	248	266	51	102,601	0.19	19,672	259
**5–15**	52	5	209,082	0.10	20,104	25	146	30	275,988	0.21	56,710	53	201	36	376,001	0.18	67,343	53
**15–50**	123	18	426,956	0.15	62,481	29	891	252	550,382	0.28	155,664	162	1040	592	670,309	0.57	381,561	155
50–64	61	29	71,160	0.48	33,830	86	358	125	80,731	0.35	28,188	443	471	148	97,207	0.31	30,545	485
>65	46	12	21,968	0.26	5731	209	189	77	37,400	0.41	15,237	505	297	105	41,507	0.35	14,674	716

*Note*: Number of persons hospitalized with severe acute respiratory infection (SARI). (A) Number of persons hospitalized with influenza‐associated severe acute respiratory infection (SARI). (B) Total population of catchment area Islamabad sentinel site. (C) Proportion of patients admitted due to influenza over total SARI hospitalization. (D) Estimated catchment area population. Incidence rate due to influenza‐associated SARI over estimated or proportion of population/100,000 population. The red colour text shows highest incidence rate/100,000 population of influenza‐associated SARI cases were in <1 year of age followed by 1–2 years of age group.

However, during 2018, the estimated incidence rate were highest with 505/100,000 cases in age group 65 years and above (95% Cl: 475–535) followed by 443/100,000 in age group 50 to 65 years (95% Cl: 403–473) and lowest of 53 in age group 6 to 15 years (95% Cl: 47–59) (Table 4).

A similar pattern was observed in 2019, the incidence rates were highest with 716/100,000 cases in age group 65 years and above (95% Cl: 686–746) followed by 485/100,000 in age group 50 to 64 years (95% Cl: 455–515) and lowest of 53 in age group 6–15 years (95% Cl: 47–59) (Table 4).

## 4 DISCUSSION

Although a network of nationwide sentinel site labs was purposefully established for influenza‐like illness (ILI) and SARI surveillance, the lack of availability of the administrative data in other regions hampered calculation of influenza disease burden in those regions of the country. Hence, it was decided to focus on the site where catchment area, population composition, and administrative data of other catchment healthcare facilities were available. This sentinel site FGSH is located within Islamabad City and is a tertiary healthcare facility. The site was selected based on commitment and motivation, geographic distribution, population density, availability of technical expertise, and high patient turnover.

This is the first study in Pakistan that provides estimates for hospitalizations due to influenza (incidence rate per 100,000 population). During the study period, results showed that influenza caused a significant burden in SARI patients in Pakistan which leads to 145/100,000 incidence rate for influenza‐associated hospitalizations over 3 years period. Our findings are in contrast with those from Iran, where the overall incidence of SARI caused by influenza was estimated to be 29 per 100,000 people.^11^

On the other hand, Khanh et al.,^12^ presented that SARI had influenza‐associated hospitalization rates of 134 (95% UI 119–149) per 100,000 people. Children under the age of 5 (1123; 95% UI 946–1301) and adults over the age of 65 (207; 95% UI 186–227) had the highest influenza‐associated SARI hospitalization rates per 100,000 people. This study has similar results with our study which highlighting the need for prevention and control measures, such as vaccination, in these at‐risk populations.

Although different years had variation in influenza transmission patterns, influenza viruses circulate all year round, with September in every year seeing a decline in influenza activity. Influenza virus subtypes A (H3) was the predominant strain during 2017 which was similar to findings of Iran.^13^ During 2018 and 2019 seasons, influenza H1N1pdm09 was the dominant strain followed by A (H3) and influenza B; this similar pattern of subtype circulation was observed in Egypt.^14^

The incidence rate of influenza‐associated hospitalizations was highest among those 65 years and above followed by children under 5 years. Our findings are similar with other countries, such as China,^15^ India,^16^ Oman,^17^ and Tunisia.^18^ Factors that may contribute to the hospitalization rate include access to medical care and antiviral drugs, influenza vaccination coverage, and cocirculating bacterial and viral pathogens.^19^ Hirve et al. study showed higher rate of hospitalization in adults aged above 60 years, followed by above 5 years of age.^16^

## 5 DATA LIMITATIONS

The capital city, Islamabad, has a population of 1.015 million, based upon 2017 population estimates. The sentinel site in Islamabad was selected due to availability of the administrative data and other major public sector‐owned healthcare facilities in the catchment. The data obtained are based on 1 year of SARI surveillance; therefore, seasonality is difficult to predict without multiple years of data. The administrative data of other provincial sentinel sites was not available and hence not included in the study.

The estimates of influenza in one sentinel site in Islamabad may underestimate disease burden due to the paucity of data shared by other healthcare facilities in the catchment area. Moreover, the estimation of male and female population is adjusted according to census data. Moreover, clear information on the true socioeconomic status, education level, and accessibility to Health Care Facilities (HCFs) is not known in the catchment population.

Although laboratory testing is done on all SARI samples by real‐time PCR, due to scarcity of staff, the sampling was done only on weekdays and during working hours and not on national holidays.

Some of the challenges faced are lack of persistent commitment and motivation, willingness of other healthcare facilities to share SARI data, regular provision of administrative data, sustainability of ongoing surveillance, running cost of laboratories, provincial ownership, cost of capacity building programs, retention of qualified and trained staff, and finally, fund mobilization and resource allocation.

## 6 WAY FORWARD

The present surveillance system may be strengthened provided sustainable funding for ongoing activities is available either through government or donor agencies. Other alternate resources, such as philanthropists or private sector funding, may be engaged for achievement of a common goal. Enhanced motivation, public–private collaboration, and willingness to share administrative and scientific data will enhance the outcomes of such surveillance and reduce burden of influenza. Expansion to include other respiratory viral pathogens may play a role in attracting vaccine manufacturers and tapping of additional resources. Finally, joint scientific publications in journals will be beneficial for not only informed decision making but will also help prioritize national health needs in terms of cost effective health spending.

## 7 CONCLUSION

Our study found the maximum incidence rates for influenza‐associated hospitalizations in people above 65 years of age followed by children under 5 years. In our population, 49% of influenza‐associated hospitalizations was accounted for by A(H1N1)pdm09 influenza virus. In 2017, other seasonal influenza A viruses accounted for 87% of the burden. In contrast, during 2018–2019, A(H1N1)pdm09 predominated and resulted in high hospitalization rates. Influenza B‐associated hospitalization rates were almost twofold higher during 2019 as compared with 2017 and 2018. Based on these findings, we understand that influenza accounts for a significant proportion of respiratory morbidity and hospitalization. These estimates from one sentinel site can serve as a first step towards accurate estimation of national influenza‐associated disease burden. Furthermore, it is necessary to test for other respiratory pathogens such as respiratory syncytial virus, human metapneumovirus, and rhinoviruses in influenza‐negative samples for better respiratory disease burden estimation. Reliable disease burden estimates will assist public health managers and policy makers to prioritize allocation of health resources for surveillance, prevention and control, and plan immunization strategies for the high‐risk groups.

## AUTHOR CONTRIBUTIONS

**Muhammad Salman:** Conceptualization; funding acquisition; resources; supervision; writing—review and editing. **Nazish Badar:** Conceptualization; data curation; formal analysis; investigation; methodology; project administration; software; supervision; validation; writing—review and editing. **Aamer Ikram:** Funding acquisition; project administration; resources; supervision; validation. **Nadia Nisar:** Data curation; formal analysis; investigation; methodology; software; validation; visualization. **Umer Farooq:** Formal analysis; visualization; writing—review and editing.

## CONFLICT OF INTEREST STATEMENT

There is conflict of interest of author and any coauthor of this article.

### PEER REVIEW

The peer review history for this article is available at https://www.webofscience.com/api/gateway/wos/peer-review/10.1111/irv.13125.

## Data Availability

All relevant data are within the paper tables and figure files.
